# Scientific and regulatory evaluation of mechanistic *in silico* drug and disease models in drug development: Building model credibility

**DOI:** 10.1002/psp4.12669

**Published:** 2021-07-13

**Authors:** Flora T. Musuamba, Ine Skottheim Rusten, Raphaëlle Lesage, Giulia Russo, Roberta Bursi, Luca Emili, Gaby Wangorsch, Efthymios Manolis, Kristin E. Karlsson, Alexander Kulesza, Eulalie Courcelles, Jean‐Pierre Boissel, Cécile F. Rousseau, Emmanuelle M. Voisin, Rossana Alessandrello, Nuno Curado, Enrico Dall’ara, Blanca Rodriguez, Francesco Pappalardo, Liesbet Geris

**Affiliations:** ^1^ EMA Modelling and Simulation Working Party Amsterdam The Netherlands; ^2^ Federal Agency for Medicines and Health Products Brussels Belgium; ^3^ Faculté des Sciences Pharmaceutiques Université de Lubumbashi Lubumbashi Congo; ^4^ Norvegian Medicines Agency Oslo Norway; ^5^ Biomechanics Section KU Leuven Leuven Belgium; ^6^ Virtual Physiological Human Institute Leuven Belgium; ^7^ Department of Drug and Health Sciences University of Catania Catania Italy; ^8^ InSilicoTrials Technologies Milano Italy; ^9^ Paul‐Ehrlich‐Institut (Federal Institute for Vaccines and Biomedicines) Langen Germany; ^10^ European Medicines Agency Amsterdam The Netherlands; ^11^ Swedish Medical Products Agency Uppsala Sweden; ^12^ Novadiscovery Lyon France; ^13^ Voisin Consulting Life Sciences Boulogne (Paris) France; ^14^ AQuAS ‐ Agency for Health Quality and Assessment of Catalonia Catalonia Spain; ^15^ Exploristics Belfast UK; ^16^ Insigneo Institute Sheffield University Sheffield UK; ^17^ Department of Computer Science British Heart Foundation Centre of Research Excellence University of Oxford Oxford UK; ^18^ GIGA In silico Medicine Université de Liège Liège Belgium

## Abstract

The value of *in silico* methods in drug development and evaluation has been demonstrated repeatedly and convincingly. While their benefits are now unanimously recognized, international standards for their evaluation, accepted by all stakeholders involved, are still to be established. In this white paper, we propose a risk‐informed evaluation framework for mechanistic model credibility evaluation. To properly frame the proposed verification and validation activities, concepts such as context of use, regulatory impact and risk‐based analysis are discussed. To ensure common understanding between all stakeholders, an overview is provided of relevant *in silico* terminology used throughout this paper. To illustrate the feasibility of the proposed approach, we have applied it to three real case examples in the context of drug development, using a credibility matrix currently being tested as a quick‐start tool by regulators. Altogether, this white paper provides a practical approach to model evaluation, applicable in both scientific and regulatory evaluation contexts.

## INTRODUCTION

In healthcare‐related academic and industrial research and development, modelling and simulation (M&S) (*in silico*) approaches are combined with other advanced scientific tools to support innovation for better understanding physiology, pathophysiology, complex diseases and effects of medical interventions. The value of *in silico* methods in drug development and evaluation has been demonstrated repeatedly and convincingly.^1^, ^2^ Computational models have evolved from being a possible alternative to other data sources (*e*.*g*. clinical trials), to being an unmissable must for development of new medicines, drug maintenance on the market and extension of indications for existing drugs (*i*.*e*. their repurposing in completely new indications). From additional or descriptive evidence used in some sparse cases, digital evidence (as generated by *in silico* models) is now included in almost all regulatory submissions. In many cases *in silico* models constitute the key source of evidence in drug development programs and related regulatory submissions (*e*.*g*. in case of extension of indications to children based on extrapolation). The term model‐informed drug development (MIDD) is often used to describe the approach of using models to inform drug development (see e.g. 3, 4, 5, 6, 7 ).

Modelling and simulation is a rapidly evolving area in terms of both technologies and application field. The latter are expanding beyond the description of drug exposure, towards the dynamic description of complex drug effects and disease subtypes and progressions. With the combination of increased uptake and expanding technologies, it is essential to have clarity and consensus in the *in silico* community on the most appropriate tools for *in silico* model evaluation. Therefore, the aim of this white paper is to present a high‐level framework that can guide the process of the evaluation of models and simulations in a holistic and comprehensive manner. The suggested framework can be seen as a generic umbrella that can be used irrespective of model and simulation technology, by guiding the process of their evaluation rather than providing the technical content and requirements. In making the process of their assessment explicit, it facilitates better informed dialogue between stakeholders, which currently is a challenge as modelers often may overwhelm other domain experts with technical details.^8^, ^9^

Ideally, *in silico* tools should be endorsed by all the relevant stakeholders, including academia and industry researchers, regulators, payers (HTA), healthcare professionals and patients.^10^ An important aspect of this approach is that, principally, the quality standards for establishing model credibility should be driven by the scientific question to be addressed by the modelling and simulation exercise, the context of use and the risk involved rather than by the type of sponsor (academia, industry, health care etc.), or whether the use is early experimental development *versus* regulatory use. Of note, The EU HMA/EMA strongly encourages academic consortia to engage actively in the process of drug development and regulatory science and several schemes are put in place at EMA level to facilitate this process.

A common language and approach can give us an environment that could permit establishing the credibility of *in silico* models and their adequate use in an objective and consistent manner across the various modelling and simulation technologies and applications. In the context of drug development, the credibility framework can be seen as complementing the stepwise model building and validation that today is standard for drug development. One could say that MIDD is this step wise building and consolidation of the *in silico* backbone of knowledge on the medicinal product coupled with an open dialogue between developers and regulators on finding potential applications for modelling and simulation to inform the development and approval.^3^, ^4^, ^5^, ^6^, ^7^

State‐of‐the‐art papers, tutorials and regulatory guideline documents exist for methodological validation and reporting of Quantitative Structure‐Activity Relationship (QSAR) methods ^11^, ^12^ as well as some pharmacometric approaches, such as population‐pharmacokinetics (popPK), pharmacokinetics/pharmacodynamics (PK/PD), and dose/exposure‐response (DER) models.^13^, ^14^, ^15^ In contrast, regulatory guidance on mechanistic models (models developed starting from mechanisms, see Table in Section 2 for a full definition) is scarce. The EMA and FDA physiology‐based guidelines on pharmacokinetics (PBPK) models can be cited as pioneers in this domain.^16^, ^17^ With the aforementioned increase in model technologies used in drug development, there is an unmet need to provide an environment that would permit establishing the credibility of mechanistic *in silico* models and their adequate (regulatory) evaluation in a consistent manner.

This white paper aims to provide input on rigorous scientific and regulatory evaluation strategy for the expanding range of *in silico* technologies currently used in drug development. We will present a high‐level framework, inspired by the ASME V&V40 for medical devices,^18^ that could guide the evaluation process of models and associated simulations in a holistic and comprehensive manner without necessary focusing on very technical and specific aspects related to different applications or types of models (these topics will be covered in future communications). To properly frame the required credibility building activities, concepts such as context of use, regulatory impact and risk‐based analysis will also be discussed. An overview of the relevant *in silico* terminology used throughout this paper will be provided. The steps of the risk informed credibility assessment will be presented, framing the required verification and validation activities, and concepts such as context of use, regulatory impact and risk‐based analysis will be introduced and discussed in the paper. During model building and validation activities, data from different sources are often used. Therefore, considerations of model predictions relative to data from other sources, and adequate uncertainty quantification and mitigation are some of the key and challenging steps. To illustrate the feasibility of the proposed approach, we have applied it to three real use cases use cases in the context of drug development, showing the variety of technologies and applications that are covered by the framework proposed in this white paper.

## TERMINOLOGY

One main challenge in the communication between relevant stakeholders is the terminology associated with the *in silico* methodology. A uniform and widely adopted consensus terminology is lacking amongst all *in silico* developers, assessors and users. Scientists reporting their results to their community or sponsors presenting their submissions to the regulators often use terms with a different understanding than the one expected by their audience (be it reviewers, readers, regulatory assessors or others). The need for at least a mutual understanding of the *in silico* terminology used by each party is thus of utmost importance as it directly affects the communication efficiency between stakeholders.

For example, the term *qualification* is more commonly used in the EU drug regulatory space (EMA) ^19^ to design requirements for a model to be considered fit‐for‐purpose whereas terms *verification and validation* will rather be encountered in the medical device developers’ space to discuss model adequacy requirements.^18^ This can be explained in part by the fact that drug regulators are used to risk‐based decision making in a multifactorial context and will aim to qualify models for a particular context of use given the established regulatory impact (with as bottom line: the perfect model does not exist). In contrast, for medical device, the mechanisms (*i*.*e*. physical laws) are better defined: developers can define tools to assess the adequacy of their model predictions. Another example pertains to the terminology used to indicate a specific type of model. Clinical pharmacologists will mostly use the term *pharmacometric modelling and simulation* while engineers will use the term *in silico models* to indicate almost exactly the same concept: a set of mathematical equations and/or computer algorithms that can be used to predict drug effects and/or health outcomes in different scenarios.

In our opinion, even if very convenient, the use of the same terminology becomes less critical when there is a common understanding of what is meant by specific terms used by each party. Given the continuous expansion of the modelling application domain, harmonization of language should not be the first target. The first target should rather be the understanding of nuances, similarities and differences in the terms used for different applications and by different stakeholders. It is important to understand which terms are interchangeable or overlapping, and which ones have different meanings when used in different settings. When different stakeholders communicate (*e*.*g*. scientists publishing a paper, sponsors meeting regulators), it is paramount that the most important terms are clearly defined at the onset, so misunderstandings are avoided. This effort may be supported by leveraging existing organized ontologies relative to modelling of biological processes (*e*.*g*., Mathematical Modelling Ontology, Systems Biology Ontology).^20^, ^21^ Consistency across requirements for model acceptability by assessors/reviewers will strongly depend on the appropriate use of terms by developers/reporters and their adequate understanding by users/ readers/reviewers/assessors. This is particularly important for *in silico* models requiring multidisciplinary expertise at the intercept of several scientific fields. To set the example, we start this white paper with a glossary (Table 1), clarifying our understanding of the most commonly used *in silico*‐related terminology in the field of drug development and evaluation. Ideally, this list should be expanding and regularly updated based on new application domains of modelling and simulation.

**TABLE 1:** Definition of key terms related to computer modelling and simulation in drug development

Biological system	Complex ensemble of entities of a population or an individual that are interdependent and function as a whole. The entities and the limits of the defined system itself may be of various scale of organization (population, individual, physiological system, organ, cellular, molecular, etc.).
*In silico* models	Abstract and simplified representation of a biological system, composed of a set of rules or algorithms describing the system’s behaviour, implemented and studied computationally. According to the mathematical nature of the rules, the behaviour of the system can be studied over time and/or space and quantitatively or qualitatively. The term in silico refers to the computational nature of the model and discerns it from its in vitro and in vivo counterparts.^22^ In the context of drug development, the set of mathematical rules and algorithms are typically used to predict drug effects and/or disease outcome in different scenarios. Thus, it is a generic term referring to a broad scope of computational models such as Quantitative Systems Pharmacology, system medicine and physiology‐based multi‐scale multi‐physics models.^23^, ^24^, ^25^ From a methodological point of view, in silico models may be found at any level of the spectrum ranging from fully data‐driven to fully mechanistic models (cfr definitions below)
*In silico* clinical trials	Class of trials for pharmacological therapies ^26^, ^27^, ^28^, ^29^ or medical devices based on modelling and simulation technologies. Such trials produce digital evidence that can serve in complement to or replacement of *in vivo* clinical trials for the development and regulatory evaluation of medical therapies.^30^, ^31^
Data driven models ( black‐box models, phenomenological models	Models developed from observations or data with the aim of reconstituting a set of rules explaining those data. These models can be developed using statistical, mathematical and/or computational methods including bioinformatics, machine learning and artificial intelligence. This type of models is built to match the observation content of the data but the resulting rules do not necessarily correspond to real, physical or tangible mechanisms, which makes it more difficult to interpret, hence the term of black‐box model.^22^
Mechanistic models (white box models, hypothesis‐driven models)	Set of theoretical rules and algorithms based on known mechanisms expected to reconstitute observed behaviours. Consequently, the rules describe known or hypothesized mechanisms in a lower scale of organization and the model read‐out often regards an emerging behaviour at a higher scale of organization. This type of model is essentially hypothesis‐driven and allows to test the validity of the underlying mechanisms, and to explain an observation, hence the term of white box model.^22^ Most mechanistic models contain phenomenological elements because of abstractions that are made, e.g. a mechanistic model at the tissue level does not capture the mechanisms at the cellular or subcellular level. Nevertheless, when the model is built around the known mechanisms we use the term mechanistic despite the presence of some phenomenological elements,
Agent‐based models	Agent‐based models (ABM) are an effective approach for modelling discrete, autonomous agents such as cells or bacteria.^32^
Artificial Intelligence (AI)	In the field of *in silico* modelling, AI is a set of technologies that have an adaptive and anticipatory capacity to deal with a defined problem while showing a certain degree of autonomous learning and improvement in solving the problem in question. The scope of technologies belonging to AI is very broad and includes, for example, machine learning, deep learning, etc. The capacity to learn for an algorithm may arise from different processes such as supervised or non‐supervised learning and reinforcement learning.^33^
Model‐Informed Drug Discovery and Development	Quantitative framework for prediction and extrapolation, centred on knowledge and inference generated from integrated models of compound, mechanism and disease level data and aimed at improving the quality, efficiency and cost effectiveness of decision making.^4^
Pharmacometrics	Pharmacometrics is the branch of science concerned with mathematical models of biology, pharmacology, disease, and physiology used to describe and quantify interactions between xeno biotics and patients, including beneficial effects and side‐effects resultant from such interfaces.^29^ Related activities encompass developing and applying mathematical and statistical models to characterize, understand, and predict a drug’s PK/PD and biomarker‐outcome behaviour.^34^
Population PK (PopPK) and Pharmacokinetic / Pharmacodynamic (PK/PD) models	PopPK is the study of pharmacokinetics (i.e., time course of concentration at a certain dosing regimen) at the population level, in which data from all individuals in a population are evaluated simultaneously using a nonlinear mixed‐effects model.^35^ PK/PD‐modelling links dose‐concentration relationships (PK) and concentration‐effect relationships (PD), thereby facilitating the description and prediction of the time course of drug effects resulting from a certain dosing regimen.^36^
Physiologically Based Pharmacokinetic (PBPK) models	PBPK models estimate the PK profile or exposure in “a target tissue or organ after a drug dose by taking into account the rate of absorption into the body, distribution among target organs and tissues, metabolism, and excretion”.^37^
Systems medicine models	This type of *in silico* model aims at studying a pathophysiological system by focusing on various possible biological scales. The scale may vary from the genetic and signalling pathways to cell‐cell communication, processes at tissue level and clinical outcomes. Such models often focus on a specific disease and attempt to predict the effect of a specific type of treatment, sometimes on a defined branch of the population. This defines the model's context of use.^38^
Quantitative Systems Pharmacology (QSP)	QSP is broadly defined as an approach to translational medicine that combines computational and experimental methods to elucidate, validate and apply new pharmacological concepts to the development and use of small molecule and biologic drugs. QSP will provide an integrated “systems level” approach to determining mechanisms of action of new and existing drugs in preclinical and animal models and in patients.^39^
Model uncertainty	A certain amount of contingencies and inaccuracies may arise from the model predictions/simulations and resulting decisions. These uncertainties may be due to the model structure (assumptions), parameters and/or the inputs.^40^
Model uncertainty quantification	Characterization of the model uncertainty with quantitative metrics. It assesses how much the outcome of the model is impacted when some part of the system or some inputs are changed or not precisely known. By systematically identifying the sources of uncertainty, characterizing their probability distribution and analysing their impact on the model's outputs of interest, the evaluation process ensures that the uncertainty's impacts on the model predictions are understood and controlled.^32^, ^41^
Historical data / Legacy data	Data previously collected in a relevant context but for a different purpose. Historical data, when appropriate for the context of use and of sufficient quality, can be used for validation of new models.^42^
Good simulation practice	In analogy to the ICH Good Clinical Practice or the OECD Good Laboratory Practice, GSP could be a quality standard for the designing, implementing and reporting of in silico trials in the context of the development and regulation of medical treatments. When established in concertation with the proper authorities, compliance with the GSP standard could ultimately provide public assurance that the digital evidence generated by in silico technologies is credible.^40^, ^41^

## CURRENT REGULATORY APPROACH FOR *IN SILICO* MODEL EVALUATION IN THE CONTEXT OF DRUG DEVELOPMENT AND EVALUATION

Modelling and simulation takes its roots in academia and has been embraced by sponsors (including drugs’ commercial sponsors) who have rapidly understood the economic potential of this approach. When developing and assessing the models, academic researchers have emphasized the innovation and the scientific (technical) value of the models, focusing on different requirements to define their acceptability (*e*.*g*. mathematical, statistical, computational, pathophysiological, pharmacological). This has led to a multiplication of tools for model evaluation, covering a wide range of methodologies for mathematical and statistical model evaluation, independent/external validation, scientific ((patho)physiological/pharmacological) plausibility of parameter estimates, etc.^42^ Some model evaluation tools were developed specifically for the purpose of transfer to clinical applications.^43^

However, these tools still have a long way to go in terms of implementation and general uptake and in terms of rigor in their implementation which currently can be variable and unbalanced (*i*.*e*. very strong claims are often made by developers with poor reporting and/or a weak verification/validation process). In this white paper, we will discuss the different available evaluation tools that we believe could be of interest for model evaluation (see section 9).

Drug developers and regulators have hitherto more focused on the application itself (drug) and the specific question that can be addressed by the models. Model development and evaluation are proposed in well‐defined contexts of use that take into account the regulatory impact (*cf*. section 7) and the consequence of model inadequacies for the relevant decision making at the level of the patient and public health, making some models acceptable for some contexts but not for others. This entails the necessity of pre‐defining the requirements for model acceptability in an application‐wise manner, irrespectively of the specific model technology used (*e*.*g*. empirical statistics *vs*. two‐stage approach *vs*. empirical models *vs*. PBPK(‐PD), SM/QSP models *vs*. agent‐based models). This also shows the need for understanding the actual value (strengths and limitations) of the proposed/available tools for model evaluation, their applicability to various model technologies and their relevance for the scientific question(s) of interest.

The regulatory evaluation of models is ideally supposed to be in line with the latest scientific knowledge in the field. While sponsors have the full liberty (and are encouraged to be as innovative as possible) in the choice of the approaches for evidence generation to support their claims, the regulatory assessors’ role is to ensure that the proposal is scientifically sound and valid given the context of use, the regulatory impact, and given pre‐established standards. For the interest of patients and public health, the regulators should always push the sponsor to provide the highest level of evidence and quality of modelling data. Although driven by regulators, establishment of standards should be a joint effort between all the stakeholders involved in the process (*i*.*e*. regulators, industry, academia, patients, healthcare professionals, HTA and payers). The current gaps and challenges encountered in *in silico* model reporting and evaluation likely affect the interactions between sponsors and regulators and, in some cases, this can delay drug market access for new drugs that patients need.

From a practical perspective, the regulators are expected to provide some guidance to sponsors on the presentation of their model development and evaluation results for optimal communication. At EMA, a range of regulatory procedures exists to interact and dialogue with sponsors on case‐by‐case basis, including for M&S related aspects. These procedures include Innovation Task Force (ITF) meetings, scientific advice protocol assistance and qualification advice/opinion.^44^, ^45^, ^46^

The ongoing Model‐Informed Drug Development (MIDD) Pilot Program at the FDA is a very good example of how modelling related questions and context are fine‐tuned to ensure clarity and set expectations, thanks to regular interactions between sponsors and regulators.^47^ Additionally, reference documentation is available such as Q&A documents, concept and reflection papers and specific guidelines.^9^, ^16^, ^48^, ^49^

It should however be noted that, apart from the PBPK guideline, none of these guidance documents refers specifically to the *in silico* models beyond the pharmacometric family. Hence, there is an unmet need for regulatory guidance for *in silico* model evaluation also including models built with other technologies (*e*.*g*., agent‐based models) in the context of drug development and evaluation, a need shared by regulatory agencies worldwide. One possible reason explaining this absence of specific guidance could be the lack of focus within the scientific community on creating evaluation tools able to meet regulatory scrutiny. Another reason could be that historically, the number of regulatory submissions including these types of models is rather low compared to pharmacometric models, which is a vicious cycle as without proper guidance sponsors are reluctant to include digital evidence into their dossier. Importantly, the framework presented in the white paper, including regulatory impact aspects, can contribute to the consistency of regulatory assessment across world regions. The authors therefore strongly support the idea that this subject constitutes a good candidate for a consensus approach (*e*.*g*. through a new ICH guideline).

## POINTS TO CONSIDER FOR EVALUATION OF MECHANISTIC *IN SILICO* MODELS

**FIGURE 1:**
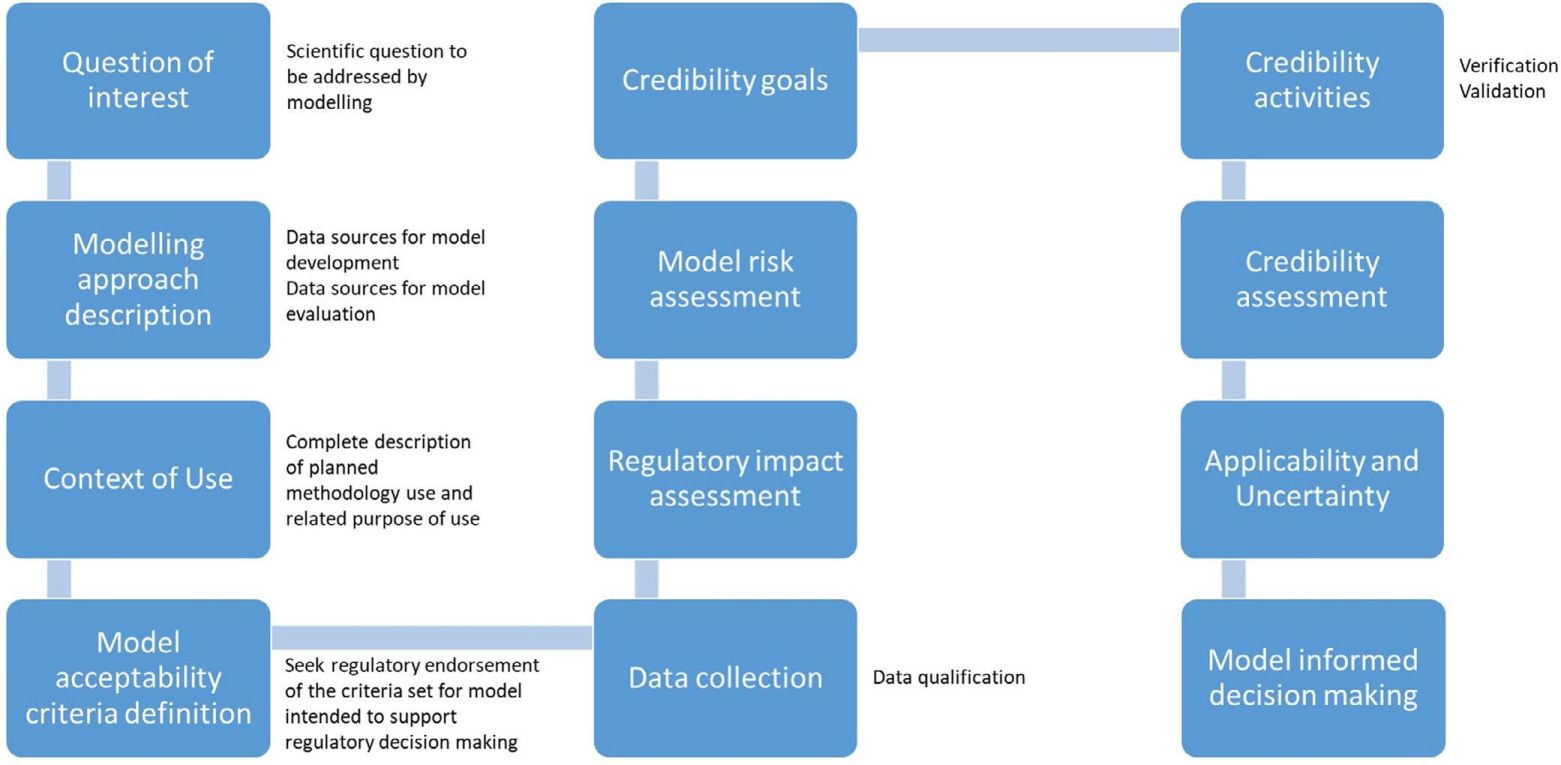
In silico Model Process flowchart

While this paper is not intended to provide a definite recommendation for evaluation, it is the authors’ conviction that a framework to support the *in silico* model evaluation rationale is needed, similarly to what is proposed in the ASME V&V40 for medical devices.^18^ The steps listed below and elaborated in the following sections are considered essential in the process of model evaluation, being it for communication between *in silico* scientists (*e*.*g*. publications in scientific journals, communications in meetings) or for regulatory submissions. A flowchart of the different steps is provided in Figure 1.

1. Description of question(s) to be addressed and Context of Use (COU) (section 5)
2. Definition of model acceptability criteria for proposed question(s) and COU (section 6)
3. Description of model influence and/or regulatory impact (only for regulatory submissions) (section 7)
4. Risk‐based analysis of decision consequence (section 8)
5. Description of model credibility activities (model verification & validation activities) (section 9)
6. Applicability and Uncertainty (section 9)
7. Model‐informed decision making

Ideally, these points should be defined at planning stage and included in a modelling and simulation plan, *i*.*e*., a document in which relevant assumptions, input data, implementation steps and output are clearly presented along with mitigation measures. This can constitute the basis for continued dialogue and interaction with regulators. To illustrate the points made below, we will work with three use cases, examples of *in silico* models in the context of drug development. A full description can be found in section 11.

## QUESTION OF INTEREST AND CONTEXT OF USE

The scientific question(s) to be addressed by the modelling exercise need(s) to be clear and well described. Each question should be stated separately in case several questions would be addressed by the proposed model. Once all the questions are well identified, the next step is to describe the proposed modelling approach to answer each question, including the data sources for model development and evaluation. It is critical to ensure that the modelling and simulation final output really addresses the scientific question. According to the EMA current policy, the Context of Use (COU) is considered to be the full, clear and concise description of the way the methodology is to be used and the related purpose of the use.^9^ The COU is a critical reference point for the regulatory evaluation of any qualification application as will become clear by the frequent referral to the COU in the following sections.

Examples of questions of interest are given below. These examples show that questions of interest are not limited only to interactions between drugs or their pharmacodynamics effects or even the interaction between a drug and organs/diseases. They can extend to the demonstration of clinical utility in the wider population after market uptake.

● What is the effect of enzyme Z inhibition and/or induction on drug X as a victim drug?
● What is the effect of drug X as perpetrator on other drugs via enzyme Z inhibition +induction?
● What is the relationship between drug X systemic concentrations and pharmacodynamic effect A at cellular/organ level?
● What is the relationship between drug X systemic concentrations and clinical efficacy response A?
● What is the relationship between state of cell A/organ B at time of treatment and the effect of drug X systemic concentration on the clinical efficacy?
● How will the drug X perform in patient populations that were excluded from clinical trials?
● How long does the drug X have to be administered to achieve the desired effects?
● What is the lowest dose at which the drug X can be given without negatively impacting treatment outcomes?
● How do the efficacy, effectiveness and safety of the drug X evolve over a longer period of time?
● How does the drug X perform in terms of patient‐relevant outcome measures?

Some examples of COU are provided below:

● Optimization of the treatment regimen for first‐in‐human studies using a QSP model (developed based on *in vitro*, *ex vivo* and/or *in vivo* animal data)
● Use of agent‐based models and related simulations for dose optimization for new therapies for infectious disease in a reference population using preclinical animal dose‐response data and exploratory clinical trial evaluating short‐term efficacy.
● Use of an agent‐based model handling sparse clinical data to predict unobserved responses/in an *in silico*‐augmented exploratory Phase 2 clinical trial
● Use of a multi‐scale multi‐physics model of a rare pathology on a virtual population to establish the effect of a specific treatment in the absence of clinical trial data in the target population
● Use of a PBPK model to establish the clinical effect of enzyme Z moderate inhibition and induction by drug X in the absence of clinical data generation.
● Application of a multi‐scale multi‐physics model on a virtual population to define inclusion criteria for a clinical trial design

Of course, the question of interest and the COU are strongly linked to one another. This can be observed clearly in the credibility matrix of the three core examples of this white paper (section 11).

## MODEL ACCEPTABILITY CRITERIA FOR A PROPOSED QUESTIONS AND CONTEXT OF USE

Model acceptability criteria need to be well established. This should be done upfront during the planning phase before the start of any data collection (see flowchart Figure 1). This includes the level of credibility and validity of models intended to support scientific claims in communications (*e*.*g*. publications) and/or regulatory decision making. For regulatory submissions, it is recommended to consider the regulatory endorsement of the set of criteria selected and the approach planned to be used for model evaluation. Different regulatory procedures and guidance documents in place should be consulted to facilitate these interactions.

Model acceptability criteria will depend heavily on the COU and on the available tools for model evaluation. For regulatory submissions, the regulatory impact also needs to be taken into account as discussed below. It is very possible that the COU needs to be refined after acceptability evaluation, especially in case an adaptive pathway approach is adopted ^50^ and HTA bodies, patients and healthcare professionals enter in an early dialogue with the regulators and industry before the beginning of Phase 2 clinical studies. In turn, it is also possible that the acceptability criteria might be expanded after new model evaluation tools become available for the proposed type of model.

In case a mechanistic *in silico* model (*e*.*g*. PBPK, agent‐based or QSP) would be used to waive a clinical study (*i*.*e*. for high regulatory impact applications) the following could be included in the acceptability criteria:

● The software platform should be qualified (as per the EMA PBPK guideline) for all the concerned metabolism pathways (section 9.1, verification)
● The mathematical adequacy of code used for the drug model should be established (section 9.1, verification)
● Parameter sources and values should be disclosed and justified for the drug model (section 9.2 validation)
● For both efficacy and safety, the impact of uncertainties in the model and their impact on the simulation results has to be discussed (section 9.2 validation).

Three examples of how model acceptability criteria can be made explicit and linked to context of use and scientific questions are provided in section 11.

## REGULATORY IMPACT

Regulatory impact is a terminology proposed for the first time by Terry Shepard ^51^, ^52^ to describe the role played by modelling data in the regulatory decision‐making. This terminology is now largely understood and widely used in the EU regulatory network. According to the regulatory impact terminology, when modelling and simulation data are only considered to play a descriptive role, because the key data for the question addressed is coming from other sources, the regulatory impact is considered to be low. However, when modelling results constitute the key source of evidence to answer the question of interest, *i*.*e*. replacing data traditionally generated in a clinical trial, the regulatory impact is considered to be high. The medium regulatory impact lies somewhere in between: modelling results are additional evidence to be complemented by other data from other sources, such as for dosing selection in a given patient (sub)population.^51^, ^52^

The concept of regulatory impact should be perceived as broader than the model influence as per the risk informed credibility assessment (ASME V&V40). Benchmarking against the current evidentiary standard is implicitly included in the regulatory impact concept. The ‘regulatory impact’ terminology is therefore interesting because it puts the *in silico* modelling data in perspective as compared to the other potential data sources (nonclinical *in vitro*, *ex vivo* and *in vivo* or clinical trials) to address the questions of interest. In addition, it implicitly compares the role of *in silico* modelling data in model‐informed drug development programs to what would have been done traditionally to address the same question without *in silico* modelling.

The stringency and the demandingness of the acceptability criteria in regulatory submissions defined in the previous section will also depend on the regulatory impact, with increasing levels of requisites/demandingness from low to high regulatory impact applications.

As illustrated in the examples in section 11, regulatory impact should in principle be explicit in each submission.

## RISK BASED ANALYSIS OF DECISION CONSEQUENCE AND MODEL RISK

After establishing the regulatory impact, the next step is a risk‐based analysis of the decision consequence. This means assessing the consequence of an adverse outcome resulting from an incorrect decision that was made based on the model. The concept of model risk has been thoroughly discussed in the ASME V&V40 standard for medical devices and can be summarised as the combination of the influence of the computational model on the decision‐making (model influence) and the consequence of an adverse outcome resulting from an incorrect decision based on the model (decision consequence). The same concept can also apply to the drug development related models as shown here. The higher the model influence and the decision consequence, the higher is the model risk. An example of such high model risk is when extrapolating efficacy and safety information from limited clinical data collected during Phase 1 and exploratory Phase 2 clinical trials.

An additional source of uncertainty in disease and drug mechanistic *in silico* models is the trial and human errors that are not easy to anticipate when creating virtual populations (*e*.*g*. dosing errors, compliance). The level of uncertainty can directly influence decisions taken based on the modelling results. It therefore becomes critical to quantify an *in silico* model's estimated uncertainties and to evaluate the implications of these uncertainties on the targeted patient populations to demonstrate the specified clinical utility. As long as the uncertainty decreases and the confidence in the new drug increases thanks to newly generated data across the clinical trials phases, the model risk lowers. An example of this is a model‐based dose selection for a confirmatory phase 3 study, based on preclinical observations and PK data in healthy volunteers and patients.^14^ Confirmatory datasets (retrospective, real‐world data and prospective), when available, can mitigate the model risk.

## MODEL CREDIBILITY ACTIVITIES

After completion of all previous steps, model credibility activities can be designed and executed. These activities start with identification of credibility goals, including desired qualitative or quantitative outcomes (*e*.*g*. pre‐specified acceptance criteria) based on scientific rationale.^18^ Credibility activities include verification of the software, the code and the calculations, validation of the model using comparator studies, and evaluation of the applicability of validation assessments to the COU. Credibility factors are the individual elements of these credibility activities, for instance the credibility activity of code verification encompasses credibility factors such as software quality assurance and numerical code verification.

The identification, design, execution and regulatory assessment of the credibility activities are general for the model assessment process. However, the specific tools and technical approaches/aspects relevant for the verification and the validation activities often differ between model types, as illustrated in.^32^ Such specific requirements are beyond the scope of this paper.

### Model verification

Verification is often termed as “solving the equations right” as opposed to validation which is “solving the right equations”. Verification is to be performed on the level of the software platform (software quality assurance), the numerical code and the calculations. The term software platform refers to a computational modelling and simulation (CM&S) software executable running on a specified offline or online computational environment characterized by its underlying operative system and hardware components. All together, these verification activities ensure that a CM&S software executable and a model are correctly implemented on the computational platform of choice, that the model is accurately solved for its intended COU, and that adequate documented evidence of the verification activities is established to enable appropriate regulatory/scientific revision.

#### Software quality assurance (SQA)

SQA ensures that the CM&S software executable is correctly functioning, and that it produces repeatable results. A relevant aspect of this testing activity is a full understanding of the CM&S requirements and specifications, the test cases, their limits, and their execution in the regression testing to ensure all the bugs, errors, and faults are addressed accordingly to their potential effect(s) on the COU and the model risk.

CM&S software can generally be classified as user‐developed software, off‐the‐shelf software (commercial or open source license), modified off‐the‐shelf software, or most frequently combinations thereof. A software developer should tailor SQA activities upon the characteristics of the software components present in the model.

For *user*‐*developed* and/or *modified off*‐*the*‐*shelf software*, SQA activities should comprise:

● Software testing, *i*.*e*. implement a manual or automated (unit test) process for specific parts of the software, where a static analysis is carried out to identify defects in the source code and in the software development environment, such as compilers and libraries called by the model.
● SQA documentation generation regarding all implemented tests, where (specific parts) and on what (specific functionalities).
● Code review on software source code, software tests and documentation review

For o*ff*‐*the*‐*shelf software* (components), SQA activities should comprise:

● Running the available benchmark verification test cases provided by the software provider.
● Documentation generation including references to the SQA procedures carried out by the software provider.

A guidance document as developed for the R statistical programming package^53^ is an example of supporting documentation to R users.

#### Numerical code verification (NCV)

The NCV process is generally carried out by the software developer and/or user. It is a process that ensures the correct implementation and functioning of the CM&S software executable and model by means of estimating the correctness and numerical error in the calculated results (including, but not limited to, the spatial and temporal convergence rates & order) as well as by performing graphical and numerical checks. When the model equations allow, this verification step can be executed with the full model (blurring the distinction between code verification and calculation verification). However, when the model becomes more complex (due to more complex geometries, equations etc.), relevant benchmark problems should be identified and used to perform code verification. See *e*.*g*.^54^ for an example of the development of simplified flow problems that can be used as standard benchmark tests for code verification for blood damage predictive models. Of note here is that, as a less stringent NCV, simulated data obtained from benchmark tests implemented with a verified code can be used for the verification of other codes.^18^

To achieve a robust NCV, the model developer should (depending on the COU):

● Compare numerical solutions returned by the CM&S software executable with exact analytical or semi‐analytical solutions provided by a verified source (*e*.*g*. provider);
● Compare numerical solutions returned by the model running on any specific computational environment other than the original settings (model numerical solutions of reference).
● Generate adequate documented evidence of the performed comparison activities and include references to the documented results from SQA verification tests conducted by the CM&S software developer

#### Calculation verification

Calculation verification encompasses estimation of discretisation errors, numerical solver errors and (human) use errors and the effect these errors have on the model results. The discretisation error arises from solving the computational problem at a finite number of spatial and/or temporal grid points. Numerical solver errors refer to errors induced by the selection of specific solver parameters. Human errors are errors introduced into the calculation by the model user in key inputs and output (*e*.*g*. typos).^18^

To achieve a robust calculation verification, the model developer could (depending on the COU).

● Analyse spatial and temporal convergence and adapt discretisation parameters or solver tolerances where necessary.
● Run a problem‐specific sensitivity analysis on the solver parameters to demonstrate that their impact on the simulation results is negligible in the scope of the envisaged model accuracy.
● Verify key inputs and outputs by either user, internal or external peer review, depending on the credibility goals for this particular credibility factor.

#### Specificities of *in silico* models for drug development

The aforementioned verification process corresponds to that of general engineering models and *in silico* models of medical devices that are heavily physics‐based. These verification actions require a well‐accepted “source of truth”. For many physics‐based models used in medical device modelling, this source is the fundamental differential equation or law that usually depends on space and time (such as the Navier‐Stokes equation to describe fluid dynamics). Models of biomedical processes rely on mechanistic knowledge, which is not always quantitative and which is not error‐free. Additionally, said processes often span multiple scales, which are intricately coupled and cannot be separated without considerable errors.^55^ Because of this, there is an increase in models that consist of multiple submodels capturing different scales (multiscale models) or phenomena (multiphysics models). In case of multiscale models, orchestration between the different space/time scales is taken care through homogenization approaches and in case of multiphysics models through transformation of properties across physics theories.^56^ For such models, the general philosophy is that credibility should be assessed at the level of an individual submodel as well as on the orchestration. In certain cases, where the homogenisation functions are also models, they too should be explicitly included in the credibility process. Another family of models that is increasingly used in the context of drug development is that of agent‐based models, which are mechanistic with at least some of the inputs being discrete. The discrete elements can range from cell state transitions (with everything else described by differential equations in a space‐time continuum) to the entire system being based on discrete rules. Verification of the continuous part of the model requires extensive verification whereas the discrete parts only require code verification as they involve algebraic calculations. However, as local instabilities might arise, a parameter exploration is warranted.

For all the aforementioned models, it is also important to ensure that model structure and related parameters are identifiable with the data used for model development. Appropriate tools should be used for this purpose.^57^, ^58^

### Model validation

As previously mentioned, validation activities are about showing whether the right equations are being solved, *i*.*e*. whether the *in silico* model is able to predict/simulate the reality of interest and the sensitivities and uncertainties of the model are clear. This starts with a clear description of the model's conceptual form along with its assumptions, ontology and input data quality. Subsequently, model validation requires the development of a validation comparator providing data to perform the evaluation. No validation can be performed without such comparator data.^32^ The assessment step then compares the prediction/simulation results with the comparator data to ascertain model credibility. Besides this direct comparison, it is equally important to estimate the uncertainty in this comparison.

#### *In silico* models, related assumptions, ontology and input data quality

Credibility factors related to the *in silico* model evaluate model form/structure (governing equations, geometry, computational domain, variables, quantities, boundary/initial conditions etc.) and model input (parameter values for model form elements). *In silico* models used in the context of drug development are mainly describing biological processes, in contrast to models used in medical devices that often depend on physics. Their form and input depend largely on non‐exhaustive and poorly measurable material (knowledge and data) even on fundamental behaviour. Consequently, there may be multiple assumptions, gaps and hypotheses necessary to construct the conceptual form and to derive (or suggest) a convenient mathematical form. These need to be clearly described in the modelling report and their impact discussed. While every model is built on hypotheses, their high quantity and potentially large impact on the output of a pathophysiology model imposes specific attention to the following points:

● A rigorous and complex knowledge management and curation (*e*.*g*. Strength of Evidence) which can evolve dynamically.
● A tight coupling between the conceptual (*e*.*g*. graph‐based on underlying pathophysiology and pharmacology) and mathematical form (equations). For instance, a unique source for equations as well as their full documentation adds more credibility in the model form.
● An emphasis on a qualitative validation assessing if best practices in the conception and documentation of the model have been implemented in addition to the quantitative validation with comparator data (thereby guaranteeing minimum standards reproducibility and quality and therefore credibility).^59^

Given the aforementioned points, the modellers should ensure and report compliance with model annotation and curation quality standards applicable for the type of model under scrutiny whenever available. Systems biology models can benefit from years of community effort into community standard ontology building.^60^, ^61^ To give an example, the use of a “scorecard” when reporting a model was recently proposed by a community effort to ensure the reproducibility of models in systems biology.^62^ As previously stated, *in silico* models of biochemical cellular and biomedical processes rely heavily on knowledge, often in the form of heterogeneous and semantic data. For that reason, important efforts must be put to thoroughly reference and annotate the model and to make sure entities and variables can be identified unambiguously, for which one can rely on the use of standard ontologies (*e*.*g*. Gene Ontology, ChEBI). That endeavor to ensure model transparency and reproducibility can be achieved by following guidelines arising from modelling community efforts such as the BioModels initiated‐effort MIRIAM (Minimal Information Required in the Annotation of Models) ^63^ or the standard SBML and CellML formats to encode models, in systems biology.^64^

A set of questions is listed below to qualitatively evaluate the credibility of an *in silico* model.

● Validation of the conceptual form: are the included knowledge, formulated assumptions validated by a biologist or a clinician in the field?
● Is the model granularity adapted to the question of interest and the context of use?
● Auditability/transparency: Is it possible to access the source justifying the model form and the parameter values?
● Uncertainty management: are the uncertainties associated with the model form and inputs and their impact on model predictions understood and controlled?
● Sensitivity analysis: Is the model sensitivity to input parameters and its impact on model predictions understood and controlled?
● Risk of tautology: is there a risk that a bias has been introduced in the model form or inputs influencing the answer into the desired way?
● Simulation design: is the *in silico* experiment design relevant to address the question(s) of interest?
● Relevance to clinical outcome: are the *in silico* model results relevant for clinical purpose?
● Relevance to the COU: are the *in silico* results relevant to the context of use and specified clinical utility?

#### Comparator data

When performing the model validation, comparator data is required. This data can come from various sources, including dedicated (*in vitro*/*ex vivo*/*in vivo*) validation experiments, or historical nonclinical, clinical trials or real‐world validation data. Additionally, this data can come from the same experiment used in model development, provided it is a different (unused) subset and is clearly defined in the approach upfront. Regardless of the source, data used to perform the validation needs to be trustworthy, meaning of good quality, and relevant to the COU. Comparator data credibility factors are related to the test sample (*e*.*g*. animal disease model) and the test conditions (*e*.*g*. drug administration). For all factors, elements such as the quantity, range of characteristics, measurements and measurement uncertainty need to be reported. The measurements performed to characterize these test samples can be used as model inputs but can, in case of quantification of the uncertainty on the input, also enable quantification of the uncertainty in the model output. Furthermore, the measurement data can be used during model evaluation to determine equivalency between model input and comparator data. All aspects of the test samples and conditions need to be investigated separately, as they will have an impact on the comparator data and hence on its usefulness to establish model credibility. Below, a non‐exhaustive list is provided of comparator data elements requiring characterisation:

● Quantity: samples sizes, number of test conditions;
● Range of characteristics: range of test sample characteristic of interest, range of test conditions;
● Measurement: rigor with which measurement data characterize each test sample, for both comparator input and output, as well as the test conditions;
● Uncertainty of measurements: uncertainty associated with tools and methods used to obtain measurements characterizing test samples and conditions.

When experimental data are reported in the literature as comparator data, most often insufficient information is available to assess the quality of said comparator data, including information on how exactly this data was established: which model systems were used, which protocols were followed, etc. This meta‐data is sometimes also referred to as the birth certificate of the data. As an example, consider the mechanical properties of arterial tissues which play an important role in models of arterial disease progression. Almost all publications describing the mechanical properties of arterial tissues will mention information such as the species and anatomical location of the tested sample, the machine used and the loading protocol. Far less publications also include information on the time between donor death and sampling, time between sampling and testing, and transport conditions of the sample even though these factors have been shown to have substantial influence on the obtained parameter values. Efforts are undertaken to ensure presence of the necessary metadata. The recently published standard ISO 21899:2020 specifies the general requirements for the validation and verification of processing methods for biological material in biobanks. ASME is looking into expanding the IT’IS (Foundation for Research on Information Technologies in Society) tissue properties database,^65^ in eight material property groups (including mechanical, thermal and electromagnetic). Additionally, at the end of 2020, a new community challenge, C^4^BIO (c4bio.eu) was launched jointly by academia and industry to develop community‐wide standardized testing protocols that include recording of the necessary metadata that will allow the data to pass regulatory scrutiny.

#### Assessment

With the previous steps completed, the accuracy of the model output can be assessed in terms of equivalency of input parameters as well as (rigor of the) output comparison.^18^ Equivalency of input parameters between the *in silico* model and comparator data is described for both type and range, with higher degrees of equivalency leading to higher credibility. Output comparison is related to a number of elements, starting with quantity – *i*.*e*. how many outputs were compared. Additionally, increased equivalency of types of output between *in silico* model and comparator leads to higher credibility. Comparison of the output can be done through visual inspection (low credibility) and direct assessment of the difference between experimental and computational results. Depending on the COU also statistical testing of predictions against random prediction null hypotheses (*e*.*g*. classification: AUROC; ranking: spearman correlation) might be warranted. To further increase credibility, uncertainties coming from experiments and computations need to be quantified and incorporated in the output. Finally, when making the comparison, the level of qualitative and quantitative agreement between quantities of interest that is deemed satisfactory needs to be in accordance with the COU (*e*.*g*. high regulatory impact requires high model accuracy and inclusion of uncertainties on both comparator and model results).

### Applicability and uncertainty

Once a model has been successfully run, then it is necessary to compare the completed activities and results with what was expected. If both are within the accepted parameters, then the model can be considered as credible; otherwise, it might be necessary to review the model or the question of interest.^66^ By evaluating the applicability of the verification and validation activities to the COU, again mindful of the model risk, an assessment of whether there is sufficient model credibility to support the COU can be made.^32^ Applicability as defined in the ASME V&V40 refers to whether the measured quantities and the application domain of the Comparator and the model are identical, which is not always the case. Pathmanathan *et al*. provide a step‐by‐step guide for analysing applicability during the validation of evidence for biomedical *in silico* models.^67^

Uncertainty is one of the critical aspects while assessing the credibility of a model. To study the model's uncertainty, it is necessary to check the uncertainty quantification (UQ) and sensitivity analysis (SA). UQ is the process of determining the uncertainty in model inputs, and then estimating the resultant uncertainty in model outputs whereas SA is the study of which inputs most affect a model output. Overall, UQ and SA test the robustness of model predictions.^67^ A critical component of any uncertainty analysis is openness of the assumptions being or not made, the tools used, and the way that results are interpreted. Educated decisions can only be made through an understanding of both the process of estimating uncertainty and its numerical results.^68^

## REMAINING GAPS AND CHALLENGES FOR THE FUTURE

The aim of the white paper is to present a high‐level framework that could guide the whole model evaluation process in a holistic and comprehensive manner without necessary focusing on very technical and specific aspects related to different specific applications or types of models. These topics will be covered in future communications. ASME V&V40 standard was indeed initially proposed for medical devices. However, this approach of establishing the credibility of a model and its associated simulations as a method to answer a scientific question of interest is general and also applicable to drug development. Some adaptation is needed to better fit to the context of drug development: of note the regulatory impact, which is one additional key point in the framework as proposed in the white paper, is not included in the ASME V&V40: The regulatory impact (which is conceptually different from the model influence) was added, to better fit to the drug development setting.

There is an increasing need for use of *in silico* models and simulations in drug development. There are for example settings where clinical data generation to demonstrate drug efficacy and/or safety is just not feasible due to ethical or practical constraints. Reliable models would offer an alternative source of evidence for drug efficacy and safety assessment and *per se* would accelerate the availability of safe and effective drugs for patients.

There is a need for rigor and ***transparency in the methods used for model development and validation*** on the one hand and their wider acceptance as a valuable source of evidence by the scientific community including academia scientists, pharmaceutical industry, regulatory bodies and HTA/payers, on the other hand. Adequate model evaluation is considered a corner stone. An environment that permit establishing the credibility of mechanistic models and their adequate regulatory evaluation/assessment in a very objective and consistent manner is currently lacking with an unmet need for that. The proposed framework can be considered as an important step toward the creation of such an environment with well established, transparent and commonly agreed criteria for establishment of mechanistic model acceptability. The methods used for this purpose need to be well described and commonly agreed. Given the novelty, the multidisciplinary nature and the relatively recent arrival in the drug development setting of these mechanistic *in silico* models, we consider that we are still in the learning phase of identifying the most appropriate tools for model verification, validation/qualification. Similar as for other quantitative tools which have been used for longer and are more widely accepted (*e*.*g*. statistical approaches, popPK, PK/PD models), it is expected that the model evaluation tools will evolve in number and in performance with the increase of the use of these types of models.

Therefore, a need exists for ***documenting the available tools***, the manners they are being used, the conditions for their adequate use and the challenges encountered. This white paper is one step in that direction, outlining a framework, similar to that of the ASME V&V40 for medical devices, emphasizing the need for transparency in the data sources and methods used, the clear link with the scientific question and the context of use, as well as the proposed acceptability of criteria. In addition, this white paper contains a glossary of key terms used in the context of *in silico* model development and evaluation. Given the sometimes‐conflicting terminologies that exist in this domain, such a glossary should be included in all communication to facilitate the communication and avoid misunderstanding during scientific review of regulatory submissions or research papers.

The ***current hurdles for larger acceptability*** of *in silico* models as a reliable source of evidence for high (regulatory) impact applications in drug development include:

1. lack of international standards and best practice documents commonly accepted by all relevant stakeholders,
2. poor communication between stakeholders to that regard, and
3. relatively slower development of regulatory science as compared to commercial solution developments.

There is currently an unmet need for ***regulatory guidance*/*best practice documents*** clearly describing standards for mechanistic *in silico* model development, evaluation and reporting considering the specificities not only in their structure, the data sources for their construction and evaluation but also in the software and algorithms used for their implementation. Ideally, this should take the format of an ICH guideline to ensure involvement of all relevant stakeholders and wider acceptability by regulators, drug developers and the scientific community. Lack of general framework of reference inevitably results in fragmentation of initiatives, development of conflicting terminologies, and difficult communication. A general framework on the other hand would ensure coordination of initiatives, harmonization of terminology and efficient communication among different stakeholders. Such a general framework for computer modelling and simulation is highly needed. Stakeholders such as the EMA, the VPH institute and the Avicenna Alliance already initiated the dialog and brainstorming on best practices for computer modelling and simulation, as illustrated in this white paper.

Besides this, the VPH institute and Avicenna Alliance are also leading an initiative focusing on Good Simulation Practice (GSP), in analogy with the Good Laboratory Practice (GLP), Good Clinical Practice (GCP) and Good Manufacturing Practice (GMP) guidelines. GSP will indeed provide a quality framework for recognition of compliance monitoring procedures. Compliance with GSP will ensure that validated models and digital data generated by *in silico* methods will be of high quality, valid and reliable.

## EXAMPLES WORKED OUT ACCORDING TO THE CREDIBILITY MATRIX

In this section, we provide three use cases of models following the above‐described verification and validation strategy. These are models that have not yet received formal regulatory approval but the developers are in various stages of interaction with regulatory bodies. After a brief summary of the model, a credibility matrix is used to provide key information on the different steps of the flowchart show in Figure 1.

EXAMPLE 1: Universal system simulator.

**FIGURE 2:**
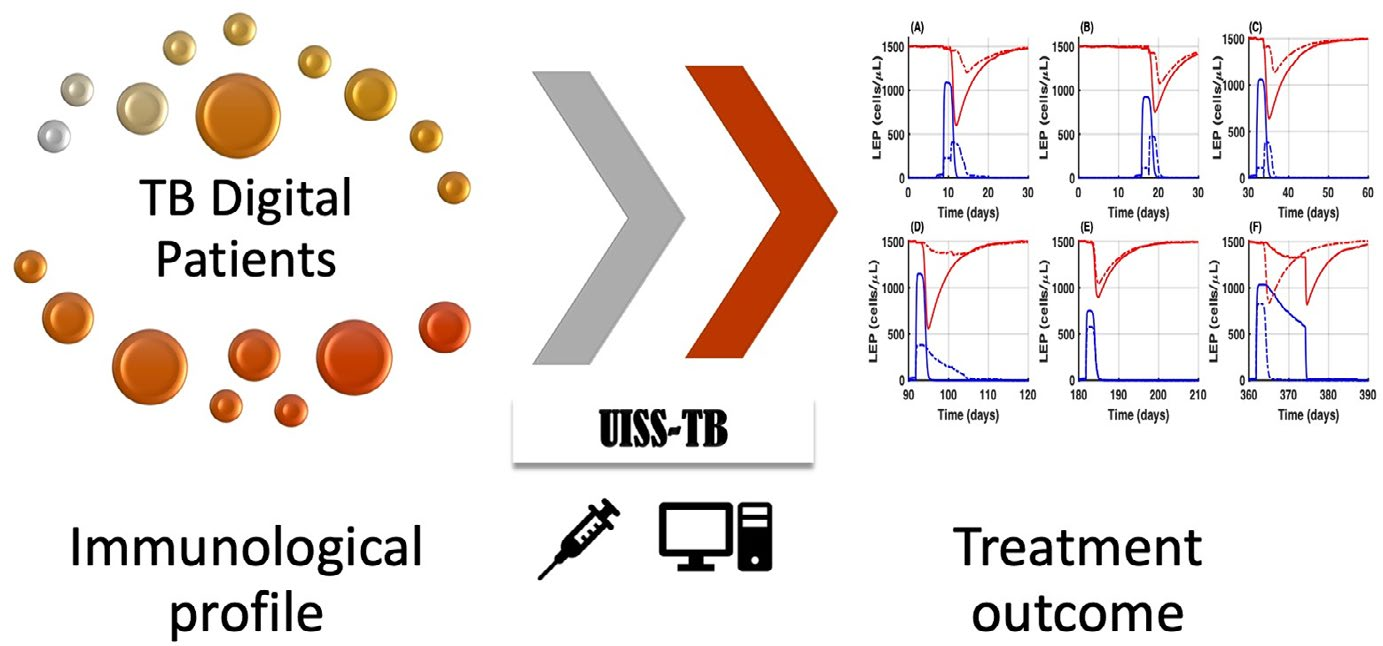
UISS‐TB predicts the dynamic of the tuberculosis course with a specific vaccine administered, suggesting possible interactions to maximize the chance of success in a personalized fashion

The Universal Immune System Simulator (UISS) is an agent‐based model of the human immune system that accounts for both innate and acquired immune response. In the past, UISS has been successfully applied to a large number of immune system disease modelling scenarios.^69^, ^70^, ^71^, ^72^, ^73^, ^74^ In preliminary studies,^75^, ^76^, ^77^, ^78^ it has been shown that the resulting simulator (UISS‐TB) could be used to simulate the relevant individual human physiology and physiopathology in patients affected by *Mycobacterium tuberculosis* (MTB) and to test *in silico* the efficacy of new vaccines against tuberculosis. (Figure 2) Moreover, UISS shows the capability of simulating the intrinsic immune system behaviour against MTB infection (eliciting or not eliciting the complete clearance of the infection or, eventually, allowing the chronic establishment of MTB reservoir inside the host due to both MTB characteristics and genetic features of the host). The key elements of the framework for UISS agent‐based model as proposed by the developer are summarized below.

**Table:** 

	**Credibility matrix**
**Drug**	Therapeutic vaccines for pulmonary tuberculosis, such as RUTI.
**Type of model**	Physiology based agent‐based model (ABM).
**Scientific Question(s) of interest (QOI)**	What is the dose‐response curve of a specific vaccine for active tuberculosis in a reference population of adults affected by *Mycobacterium tuberculosis*? What is the most optimal dose to maximize the efficacy of tuberculosis vaccine?
**Context of use**	UISS is a physiology‐ and agent‐based model of the human immune system. UISS‐TB includes a disease model component for the infection of pulmonary tuberculosis, the treatment (the therapeutic vaccine to be tested) effect component, and is run over a virtual population, representative of the target population. The aim of the model is the dose selection for confirmatory trials, with a significant reduction of the human experimentation in the phase II dose‐response trial. Data input would include: − clinical data from the phase 1 safety assessment trial − clinical data from a limited scale exploratory trial: only a single arm (*e.g*. drug‐response strains) and only two arms (a placebo group and a treatment group with a dose close to the maximum tolerated dose (MTD).
**Acceptability criteria** **(Precision level)**	**Data/input for model building** The UISS‐TB model is informed by a set of NI = 22 inputs, named vector of features (VoFs), formed by quantities that can be measured/observed in an individual MTB patient. All 22 inputs have to be considered with their admissible minimum, maximum, and average values. **Model structure and key parameters:** Model structure and parameter sources and values should be disclosed and justified for the disease and the drug model, as well as for the virtual population simulator. **Model verification acceptancy criteria** **Computation (Calculation, platform) and code verification** GitLab versioning control system will be used. The following will be monitored and results provided: For the Deterministic model − Absence of Round‐off errors − Absence of Conservation errors. − Absence of Discretisation errors. − Uniqueness: repeated deterministic runs produce identical results. − Smoothness: analyse lag correlation. − Non‐chaoticity: Lyapunov’s exponent. − Time step convergence analysis For the stochastic model verification. − Convergence and consistency analysis. o **Software Quality Assurance** GitLab QA to run regression testing, including all VV&UQ tests. Long‐term: Compliance with IEC 62304 “Medical Device Software ‐ Software Life Cycle Processes”. **Model validation activities and related acceptancy criteria** The UISS model needs to be able to model to simulate and to adequately predict the key features of patients experimentally recruited in the Phase 2 study. The UISS model needs to be able to predict the distribution of immunogenicity biomarkers at the other three follow‐up time points and compare these to those observed experimentally.
**Regulatory impact**	Medium: modelling results are additional evidence to be complemented by data from clinical trials.
**Risk based analysis of decision consequence**	In the case of UISS‐TB‐IG, an underestimation of the optimal dose might affect the efficacy of the treatment, and an overestimation might induce adverse effects. If we assume that the final decision is the marketing authorisation of the new therapeutic vaccine, the influence of the model is low for both the final efficacy component and the safety component that will rather be informed by the results of the confirmatory Phase 3 trial. For a lower‐than‐optimal prediction, we could have an increased risk of recurrence. For the higher‐than‐optimal prediction, we could have an increase of reported adverse effects. However, typical overdosing adverse effects for TB vaccines ore mild in nature (occasional muscle spasms, pain at the site of injection, etc.). Thus, also the consequence of a model error can be considered mild.
**Credibility activities results**	The credibility factors (as described in Section 9) were evaluated with overall satisfactory results Details and results of model verification activities have been previously published.^72^, ^81^
**Model informed decision**	The dose‐response relationship was characterized for efficacy of vaccine against tuberculosis that allowed optimal dose selection for the confirmatory trial.

Of note, the credibility matrix above was initially proposed by the authors and refined after discussion with regulators. As described, two related questions are to be addressed by the agent‐based model: characterization of the dose‐response relationship for efficacy and dose selection. The COU is presented with a link to the question, the data source and an analysis of clinical consequences if the model is wrong (as part of a risk‐based analysis). Regulatory impact is considered medium, model acceptability criteria are provided a reference as well for the results credibility activities implemented to meet the pre‐specified criteria.

EXAMPLE 2: Virtual Assay for Drug Cardiac Testing.

**FIGURE 3:**
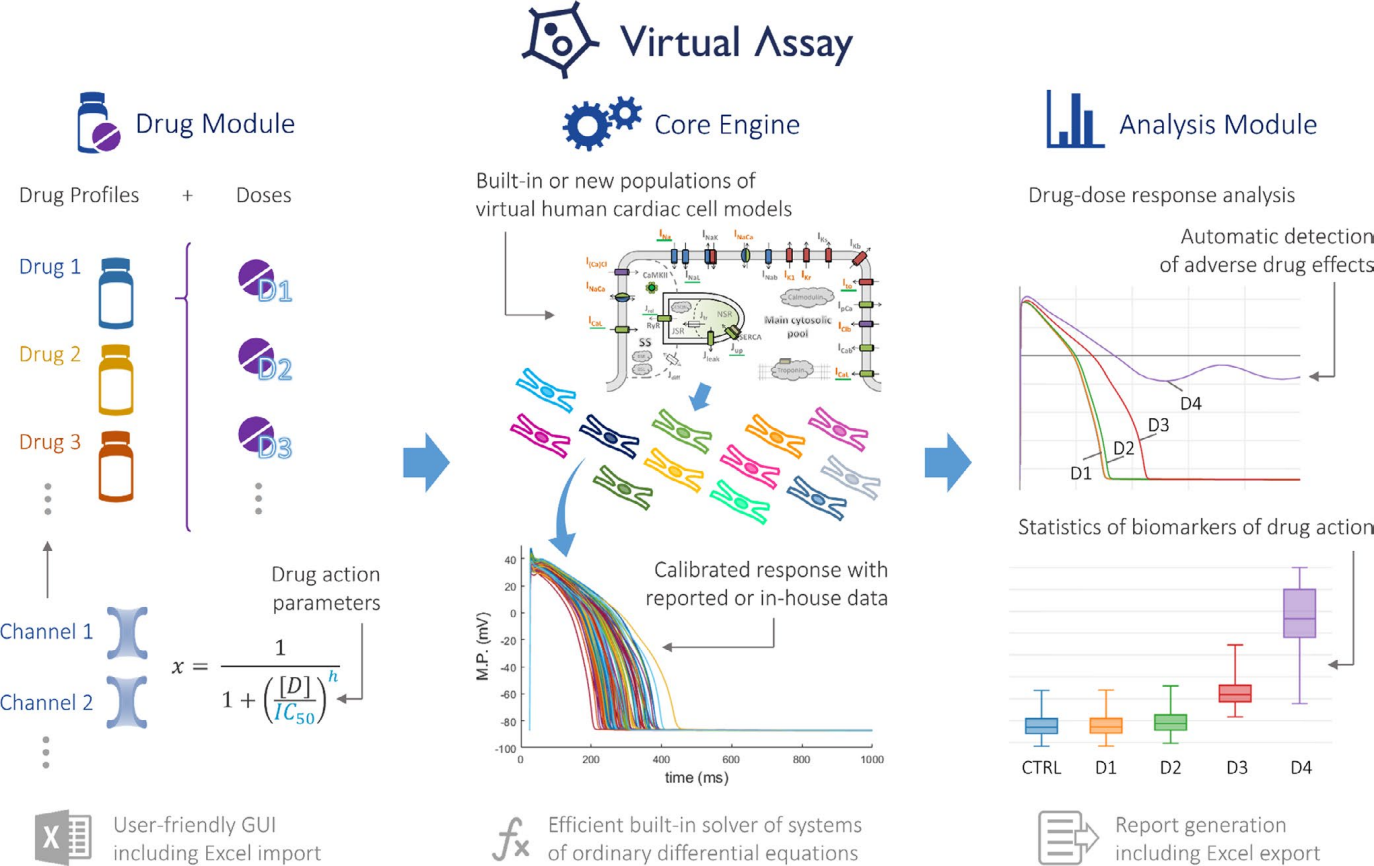
schematic overview of the Virtual Assay software platform at its main components: the Core Engine (middle), Drug Module (left) and Analysis Module (right). X: ion channel availability; h: hill coefficient; D: doses; IC_50_: half‐maximal inhibitory concentration; M.P.: Membrane Potential; CTRL: Control (no drug)

Virtual Assay is a software for running human *in silico* drug trials to augment drug cardiac testing.^79^ The core engine provides a user‐friendly graphical user interface (GUI) to efficient algorithms for the sampling and solution of populations of virtual human cardiac cell models (Figure 3). Each model in the population is characterized by a different set of ion channel parameters, with biology described in the form of systems of ordinary differential equations, producing non‐identical action potential outputs to account for variability. The Drug Module directly converts the drug action parameters for their use by the Core Engine in each of the models of the population. The Analysis Module finally generates visual reports of the conducted drug‐dose response studies, per individual model and provides statistics of biomarkers of drug action across the entire population, including the automatic detection of adverse drug effects. The key elements of the framework Virtual Assay model for drug cardiac safety testing as proposed by the developer are summarized below.

**Table:** 

	**Credibility matrix**
**Drug**	All new drugs candidates given the regulatory requirement of assessment of *in vivo* drug‐induced pro‐arrhythmic cardiotoxicity, and especially those that may be ruled out due to potential false positive signal based on hERG assays and multichannel effects.
**Type of model**	The Virtual Assay Software: human‐based cardiac electrophysiology modelling and simulation framework.
**Scientific Question(s) of interest (QOI)**	Would the drug result in risk of developing Torsades de Pointes in the human population, even in the context of positive hERG assays and multichannel effects?
**Context of use**	INPUT: *in vitro* data on drug‐induced action on ion channels (through 2 key parameters: the concentration that causes 50% of ion channel blockage (IC_50_) and the steepness of the drug response curve (Hill coefficient (h)) OUTPUT: Simulations with the Virtual Assay software categorize drugs as being safe or inducing pro‐arrhythmic cardiotoxicity in human. Decision on the potential cardiotoxicity will be informed by the simulations, combined with experimental data from animal models and potentially stem‐cell derived cardiomyocytes. Mechanistic models can be helpful to rule out a positive non‐clinical signal.
**Acceptability criteria** **(Precision level)**	**Data/input for model building:** *In vitro* data on drug action on 3 cardiac ion channels (Nav1.5, Cav1.2 and hERG). Evidence of the acceptable quality and documentation on the sources of data will be provided. **Model structure and key parameters:** The structure of the Virtual Assay software is summarised in Figure 3. The Core Engine (middle panel) generates a virtual population of human cells. A Drug Module (left panel) allows simulating the drug action on the ion channels using the input data. The Analysis Module (right panel) extracts metrics from the simulation for drug classification. Justification of model structure and parameter sources is provided in.^82^ **Model verification activities and related acceptancy criteria** **Computation (platform) and code verification** Virtual Assay has been developed in C++. Drug simulations in a modern laptop require approximately 5‐10 minutes for each drug concentration for a population of 100 cell models, and simulations are run in parallel on multiple cores. Verification of numerical scheme and code comparison has been conducted as explained in.^82^ ▪ **Software Quality Assurance** The Virtual Assay software includes documentation and benchmark verification test cases. Details on software verification are provided in.^82^ ▪ **Model assumptions and related sensitivity analysis** Sensitivity analysis is incorporated in the population of models, as this consists of using the same baseline model but with key parameters varied randomly, thus generating thousands of virtual cells. ▪ ***Numerical and graphical tools*** The Virtual Assay software incorporates a friendly interface, simulation software and visualisation of outputs. ▪ ***Uncertainty management*** The population of models approach incorporated in Virtual Assay tackles uncertainty in electrophysiology model parameters. In the case of uncertainty in input values, simulations with the most extreme cases are run and compared. **Model validation activities and related acceptancy criteria** The accuracy of drug classification using Virtual Assay was requested to be superior to the classification based on hERG alone and at least 80%. The sensitivity in the prediction of cardiac toxicity of individual drugs needs to be >60% or 70%.
**Regulatory impact**	High regulatory impact: modelling and simulation results constitute the key source of evidence to answer the question of interest, *i.e*. replacing data traditionally generated in a clinical trial
**Risk based analysis of decision consequence**	High clinical influence given the new Q&A Guidelines: impact on the decision to accept phase 1 to 3 trial designs, and also based on this model, waiver of intensive monitoring of electrocardiogram (ECG) in confirmatory trials. This is also crucial for the evaluation of cardiotoxicity in cancer drugs. Wrong model prediction/simulation could expose patients to risk of lethal arrhythmias, in following clinical trials due to cardiotoxic drugs.
**Credibility activities results**	The credibility factors (as described in Section 9) were evaluated with overall satisfactory results. Details and results of model verification activities have been previously published.^80^, ^81^, ^82^
**Model informed decision**	The drug’s pro‐arrhythmic cardiotoxicity was characterized for 62 compounds, based on their Torsade de pointe (TdP) score. Each drug could be categorized as safe or risky based on their TdP score. Subpopulations of patients at higher risk were identified for some of the drugs.

The credibility matrix above was also proposed by the authors and discussed with regulators. One question is addressed by the model and associated simulations for each tested drug: characterization of risk of developing Torsade de Pointes. The COU is presented is presented in relation to its role in the overall decision making and analysis of clinical consequences if the model is wrong (as part of a risk‐based analysis). Regulatory impact is considered high, model acceptability criteria are provided as well as a reference for the results credibility activities implemented to meet the pre‐specified criteria.

EXAMPLE 3: Myocardial physiology.

**FIGURE 4:**
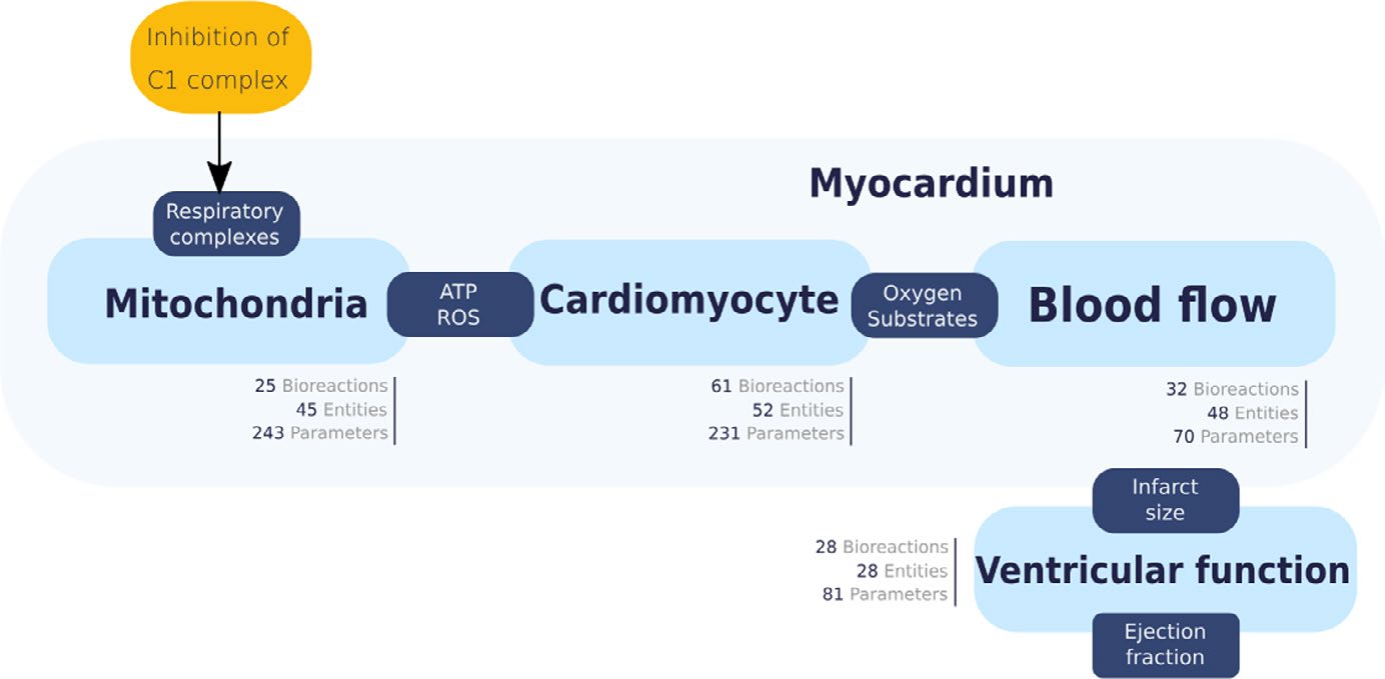
Disease Computational Model structure. Light blue rectangles represent the submodels with the associated number of parameters, variables and reactions. Dark blue rectangles represent the major connector variables shared between submodels. Myocardium submodels are duplicated throughout 10 zones to introduce a spatial discretization of the myocardium

A QSP type disease model and a Virtual Population are used to evaluate the ranges of clinical benefit through C1 modulation, and refine the target population based on response variability analysis in a Virtual Population of ST Elevation Myocardial Infarction (STEMI) treated with percutaneous coronary intervention (PCI) (Figure 4). It is hypothesized that blocking ROS production at the level of the complex 1 (C1), the beginning of the respiratory chain, will reduce damage of the overwhelming ROS production during the reperfusion and bring a relevant clinical benefit. Key features of the model as per the framework are presented below.

**Table:** 

	Credibility matrix
Investigational product	Modulator of respiratory complex 1: inhibitor of ROS production
Type of model	QSP‐type disease model: based on ordinary differential equations (ODE). The model had 625 parameters and 173 ODE.
Scientific Question(s) of interest (QOI)	What is the target population to demonstrate the effect of C1 modulation as it would have been evaluated in a classical phase 2 clinical trial?
Context of use	A mechanistic disease model describing myocardial infarction pathophysiology and effects of C1 modulation is used with a Virtual Population to identify markers that characterize responders to C1 modulation in a Phase II setting *in silico*. Data extracted from the scientific literature and preclinical *in vitro* experiments and *in vivo* experiments were used to build and calibrate the model. Individual patient data from a subset of a clinical trial dataset were used for a quantitative calibration of the clinical outcomes calculated by the model combined with a Virtual Population. The model and related simulator are proposed to support an upcoming Phase III trial design aimed at confirming the drug clinical benefit.
*Acceptability criteria*	Code verification should include the convergence analysis of all dynamics concerning space discretization of the left ventricle. As all patients will use the same space discretization, the model needs to present qualitatively the same results by predictive visual check for two discretization schemes so that it is safe to assume that inter‐patient variability is unchanged. Calculation verification was carried out by using the simulation outputs obtained with the lowest possible solver tolerances as reference solution. The error between simulated outputs and reference solutions needs to be lower than a given threshold (1%). Further quantitative acceptability criteria on the software side are model item transparency, documentation completeness and unit checking. Model acceptability is mainly assessed on the 4 outcomes with a quantitative validation based on independent individual data extracted from a previous clinical trial dataset (placebo arm, 26 patients). In our COU, the most important capability of the model is a correct prediction of a class/individual outcome based on its descriptors (and needs to be validated as such). For trial design purposes we should thus compare virtual patient classes with real patient classes and individual patient (ranks) with individual patient (ranks), respectively. According to these two requirements two precision levels have been checked for classifying ranking and patients by the model ● A response classifier model should have the capacity to identify patients with a severe outcome according to Receiver Operating Characteristic (ROC), Area Under the Curve (AUC) above 0.7 for a number of classification scenarios (similar as for any predictive biomarker). ● A response ranking model should have a significant capacity to rank individual patient's outcome severity. This capability should be tested by a suitable statistical procedure, *i*.*e*. by Spearman rank correlation significantly different from random permutation. Qualitative acceptability criteria need to be checked for validating explorative capabilities of the disease model not covered by the quantitative input (patient descriptors) output (creatine phosphokinase (CPK), troponin I (TnI), Infarct Size (IS) and left ventricular ejection fraction (LVEF)) validation. A set of credibility factors are defined including ● Model form is deemed acceptable if the conceptual form is validated by a biologist, a clinician or logical modelling; if model granularity allows the answer to the QOI; if a transparency checking is allowed in the model structure. ● Model inputs are deemed acceptable if used assumptions are listed and their impact on model prediction explored and if a sensitivity analysis has been performed ● Model is deemed relevant to the context of used if the simulation protocol is delivered prior the experiment; M&S output(s) is/are biomarker(s), a surrogate or a clinical outcome; validity domain is relevant to the COU
Regulatory impact	Medium: modelling results are additional evidence to be complemented by data from clinical trial.
Risk based analysis of decision consequence	The treatment being indicated as a complement of the first line (percutaneous coronary intervention), suboptimal patient selection will not result in harm to patients. However, it may lead to a suboptimal design for the phase 3 and a suboptimal indication for market authorisation, leading to off‐label use of the drug.
Credibility activities results	All credibility factors were evaluated: Model form evaluation ● KM validation: Acceptable (Validation by review) ● Relevance of Computational Model granularity: Good (Model granularity is adapted to the QOI(s)) ● Transparency checking: Good (Comprehensive checking) ● Model reuse: Good (The model or a part of the submodels has been reused from a different COU) Model inputs evaluation ● Uncertainty management: Poor (No uncertainty management performed yet) ● Sensitivity analysis: Poor (No sensitivity analysis performed yet) Relevance to the Context of Use ● Simulation design: Acceptable (Simulation protocol delivered prior the experiment) ● Relevance to clinical outcome score: Good (M&S output(s) is/are clinical outcome) ● Relevance to the COU: Good (Relevance of M&S output(s) of interest and validity domain to COU) The model of ischemia reperfusion was quantitatively validated on 4 outcomes. Evaluation metrics for the primary outcome (Infarct size) were the following: ● Spearman rank correlation: 0.51 ● ROC curve AUC average: 0.77 The Computational Model of myocardial ischemia reperfusion is thus validated for the anticipated use but should be completed with uncertainty analyses.
Model informed decision	Two criteria were identified to characterize optimal responders: Final TIMI flow grade above 3 and Mid or Proximal lesion location. The selection of this sub‐population doubles the clinical benefit (from 5% to 10% of average infarct size reduction). These results support a subgroup analysis with the results of a potential phase 3 clinical trial evaluating C1 modulation.

Based on the provided information in the credibility matrix by the Sponsor above, the question of interest for the Myocardial physiology model consists in the characterization of the target population to demonstrate the effect of C1 modulation. The COU in link to the scientific question is related to the optimization of the confirmatory trial design. The mechanistic model and related simulator were developed using data from the scientific literature, preclinical in vitro experiments and in vivo experiments. Individual patient data from a subset of a clinical trial dataset were used for a quantitative calibration of the clinical outcomes calculated by the model combined with a Virtual Population. Regulatory impact is considered medium, Model acceptability criteria are provided as well as the credibility activities implemented to meet the pre‐specified criteria. Risk‐based analysis and final model informed decision are also provided.

## CONFLICT OF INTEREST

The authors declared no competing interests for this work.
